# Porcelain Fused to Titanium—Advantages and Challenges

**DOI:** 10.3390/dj13090382

**Published:** 2025-08-24

**Authors:** Zlatina Tomova, Desislav Tomov, Angelina Vlahova, Gergana Kirova, Yordanka Uzunova

**Affiliations:** 1 Center of Innovative Technologies in Dental Implantology, 4002 Plovdiv, Bulgaria; angelina.vlahova@mu-plovdiv.bg; 2 Department of Prosthetic Dental Medicine, Faculty of Dental Medicine, Medical University of Plovdiv, 4002 Plovdiv, Bulgaria; 3 Research Institute, Medical University of Plovdiv, 15A Vassil Aprilov Blvd. 4002 Plovdiv, Bulgaria; desislav.tomov@mu-plovdiv.bg (D.T.); yordanka.uzunova@mu-plovdiv.bg (Y.U.); 4 Department of Chemical Sciences, Faculty of Pharmacy, Medical University of Plovdiv, 4002 Plovdiv, Bulgaria; gergana.kirova@mu-plovdiv.bg; 5 Department of Bioorganic Chemistry, Faculty of Pharmacy, Medical University of Plovdiv, 4002 Plovdiv, Bulgaria

**Keywords:** bond strength, CAD/CAM, fixed prosthetic restorations, metal–ceramics, titanium, 3D printing

## Abstract

Although dental implants appear to be an alternative for treatment of tooth loss, fixed prosthetic restorations are an irreplaceable part of oral rehabilitation. Regarding the EU directives concerning cobalt health risks, titanium alloys may be an alternative to cobalt–chromium and nickel–chromium for metal–ceramic dental restorations. The presented review briefly describes the specific properties of titanium, and the challenges met during production and use of titanium–ceramic fixed prosthetic restorations.

## 1. Introduction

In the field of Prosthetic dental medicine fixed restorations like crowns and bridges are used in cases of destruction of hard dental tissues and partial edentulism. The advantages of these types of restorations include physiological transfer of occlusal pressure through the abutment teeth to the bone, high esthetics and mechanical performance, acceptable longevity and biocompatibility [1]. Although dental implants appear to be an alternative for treatment of tooth loss, fixed prosthetic restorations are an irreplaceable part of the oral rehabilitation.

Regarding the materials used for production, fixed restorations may be divided into two main groups: full contour, produced by one single type of material, and veneered, produced by a combination of materials with different properties. The choice of the final fixed prosthetic restoration—full metal, full ceramic, or metal–ceramic—is usually made after a discussion and analysis of the most desired characteristics for each specific clinical case. Full ceramic crowns and bridges offer the best biocompatibility. However, the low mechanical resistance of lithium silicate ceramics limits their use for single crowns and small bridges in areas of low occlusal load. The lack of translucence of crystal ceramics like zirconia makes them inappropriate for cases of high aesthetic demands. The share of metal–ceramic fixed restorations is still significant due to the combination of excellent mechanical properties provided by metal infrastructure, and the good aesthetics provided by veneering ceramics.

Since the implementation of metal–ceramic production method in the middle of 20th century, the types of alloys preferred for fabrication have been changing in time. At first, noble alloys were mostly used, providing high biocompatibility and natural esthetics due to the warm color of gold [2]. Nevertheless, they were replaced by base alloys because of their superior mechanical properties and lower cost [3]. Nowadays, nickel–chromium alloys are of limited use because of cytotoxicity and nickel allergy concerns. Cobalt–chromium base dental alloys offer excellent mechanical properties, good biocompatibility, and a strong metal–ceramic bond. However, recent EU regulations might lead to restrictions on their use due to the high cobalt content and possible health risk [4]. Considering the excellent corrosion resistance and the high biocompatibility of titanium and its alloys, they may become a promising alternative to the aforementioned types for metal–ceramic dental restorations [5]. Studies show that fracture and fatigue resistance of titanium crowns are similar to those of crowns with frameworks of nickel–chromium alloys [6].

Digital technologies revolutionized dental medicine by changing both clinical and laboratory work protocols. Milling (subtractive manufacturing) and 3D printing (additive manufacturing) as a part of computer aided design/computer aided manufacturing (CAD/CAM) workflow facilitated the implementation of titanium and titanium alloys not only in the field of maxillo-facial surgery and implantology but also in everyday dental work, as they are considered perfect materials for 3D printing [7].

## 2. Titanium Alloys in Dental Prosthetics

Titanium and titanium alloys have been used in the medical field for several decades for the production of artificial scaffolds, articular joint prostheses, fracture fixation devices in orthopedics, and dental implants [7]. Titanium medical devices exhibit high corrosion resistance, acceptable mechanical strength, excellent biocompatibility, and modulus of elasticity close (but not the same) to that of the bone. They possess low specific weight and a remarkable strength-to-weight ratio, and are considered bioinert, inducing minimal adverse effects to the human body [8,9].

Titanium is an element that undergoes allotropic transformation—from hexagonal α-structure at room temperature, it transforms to body-centered cubic β-structure at 883 °C. While the stabilized α-structure provides high strength, modulus of elasticity, and hardness, the β-structure determines ductility and high temperature performance. Titanium alloys are divided into five classes—α, near α, α + β, near β, and β according to the crystalline lattice present, and different elements may be used to stabilize the desired structure and properties (Table 1) [10,11]. Standards have been created and accepted by the American Society for Testing and Materials (ASTM) regarding the composition and the indications of use of these alloys and nowadays they are classified in 38 grades [12,13].

**Figure 1:**
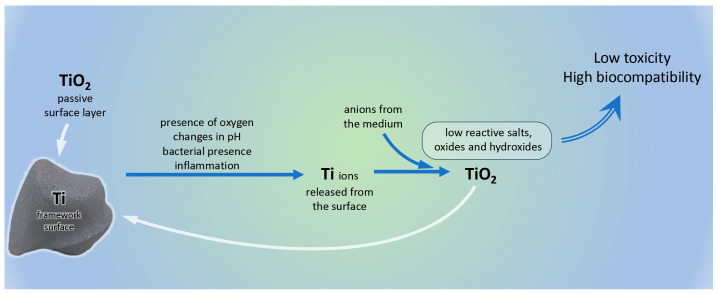
Mechanisms explaining the excellent biological properties of titanium.

The rapid spontaneous formation of surface layer of TiO_2_ leads to high corrosion resistance of titanium and its alloys [14,15,16,17,18,19,20]. However, some specific environmental factors in the oral cavity, like changes in acidity, bacteria, inflammatory diseases, tribocorrosion, and even tooth brushing and fluor-containing mouthwashes, may cause partial degradation of the passive layer [16,21,22,23,24]. As a result, ions and particles from the metal restoration are disseminated in the surrounding tissues and may be spread in distant areas of the body. Titanium ions emitted from the surface interact with anions present in the medium, forming hydroxides, oxides, and salts, which hardly react with other biomolecules [25]. The fast formation of low-reactive compounds explains the high biocompatibility of titanium and its alloys (Figure 1). Although scarce, there is evidence for pathological effects of titanium—potential change in oxidative stress levels, cellular disturbances, cases of hypersensitivity [26,27].

Corrosion resistance and overall biological safety make titanium one of the most appropriate metals for application in different areas of the human body, including the oral cavity [28].

An electronic search was completed in PubMed and Google Scholar databases for the interval 2015–2025 regarding titanium–ceramic fixed prosthetic restorations. Key words and phrases used were “porcelain fused to titanium”, “titanium-ceramic fixed restorations” or “crowns”, “titanium-ceramic bond strength”, “titanium-ceramic esthetics”, or “optical properties”. The search revealed more than 7600 articles. After initial screening, 519 articles regarding bond strength and 102 regarding esthetics were assessed. Most of the studies on titanium properties were correlated with the use of titanium in the field of implantology, investigating corrosion, osseointegration, surface implant treatment, and biocompatibility, and were excluded from further review. The aim of our article is to summarize the results of conducted research regarding titanium–ceramic fixed prosthetic restorations and to point future directions for exploration of this promising combination.

## 3. Titanium Framework Production

The conventional production method for metal ceramic prosthetic restorations involves casting of the metal alloy—the so-called lost wax casting technique. When the wax design is completed, the wax prototype of the metal framework is invested and after increasing the temperature, the melted metal alloy fills the cavity created after the wax elimination, usually under the action of centrifugal forces. The chemical properties of titanium, however, cause some restraints when this technique is used. Its affinity for oxygen at high temperatures leads to reaction with SiO_2_, which is the main refractory material used. Other types of investment materials containing different refractory compounds like MgO, Al_2_O_3_, or ZrO_2_ must be utilized for titanium casting. For preventing reaction with atmospheric oxygen, the presence of inert gases (argon, helium) must be provided for the process [29,30].

With the development and widespread of computer-aided design/computer-aided manufacturing (CAD/CAM techniques), titanium frameworks for metal–ceramic restorations can be produced by milling of titanium disks (cpTi or titanium alloys). One of the advantages of milling is the use of prefabricated cpTi (commercially pure titanium) or titanium alloys with constant composition and structure. Other advantages include lack of deformation of the final object during production and receiving a framework, precisely corresponding to the digitally created design [31]. However, this subtractive method may cause surface changes and residual stresses, affecting some properties of the restoration [32]. The amount of waste material is also a matter of concern.

**Figure 2:**
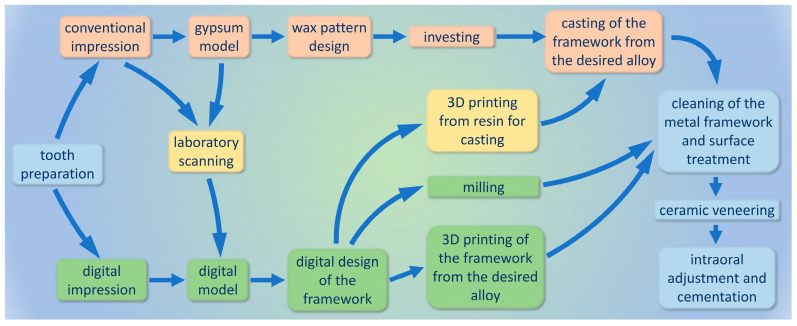
Steps of production of titanium–ceramic dental restorations: conventional production technique (orange), CAD/CAM techniques (green) and possible cross paths between the two major groups (yellow).

The implementation of 3D printing technologies like selective laser sintering, selective laser melting, and electron beam melting in the production of metal medical and dental devices allowed fabrication of more complex, customized objects. In these additive techniques powder alloys are utilized, and the final metal framework is created layer by layer using laser or electron beam power (Figure 2) [33,34].

CAD/CAM technologies require initial investment and a specific learning curve for both dentists and dental technicians. Intraoral scanning and digital design reduce the risk of deformation by eliminating the use of conventional impression and model materials, thus contributing to the fit and precision of the final restoration. Reduced clinical and laboratory time has a positive effect on the patients’ satisfaction, influencing the treatment outcome [35].

**Figure 3:**
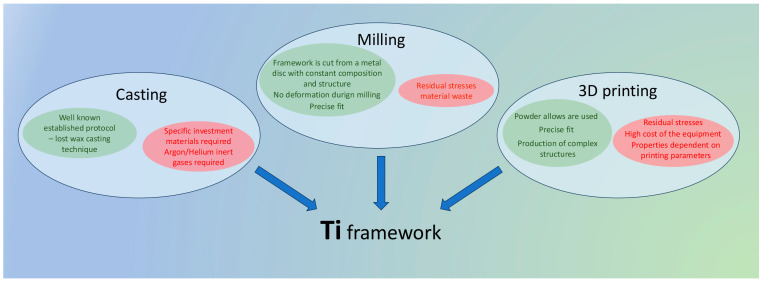
Methods of production of titanium frameworks.

Numerous studies point out that structure, surface morphology and mechanical properties are strongly influenced by the method of production (casting, milling, 3D printing) and by the specific parameters during manufacturing—type of inert gas used during casting or printing, object orientation, layer thickness and laser speed during printing, post-processing conditions [33,36,37,38,39] (Figure 3).

## 4. Metal–Ceramic Bond Strength, Surface Treatment

The compliance between the ceramic material and the titanium alloy is crucial for the longevity of metal–ceramic dental restorations. The characteristics of the surface oxide layer, coefficient of thermal expansion, surface morphology, and wettability play a significant role regarding the strength of the metal–ceramic bond. As high temperatures promote excessive oxidation of the titanium surface, low-fusing porcelain masses that comply with the properties of titanium alloys have been presented on the market with specific instructions of use given by the producers [31].

The standard ISO 9693:2019 defines the minimal requirements for metal–ceramic bond strength of 25 MPa [40]. Investigations of Uz et al. proved that the strength between titanium frameworks and ceramics is similar to that of cobalt–chromium alloys and meets the standard requirements, in cases of both milling and additive manufacturing techniques [41]. Vaska et al. came to the same conclusion [42].

However, a clinical study showing a six-year follow-up of patients with titanium–ceramic crowns revealed poor clinical performance mainly due to veneering porcelain fractures, revealing difficulties in achieving a strong and tight metal–ceramic bond [43]. Adhesion of ceramic material to titanium is likely the biggest challenge that should be overcome before wider implementation of titanium–ceramic fixed prosthetic restorations.

To modify the surface properties titanium framework may be processed mechanically by sandblasting, grinding, and polishing, or chemically and electrochemically—by etching, coating formation, micro arc oxidation, plasma spraying, and ion implantation. Required surface properties may also be achieved by the micropattern design of the surface of the titanium device using 3D metal printing technologies. All these methods lead to surface modifications which increase corrosion resistance, improve biocompatibility and osseointegration of titanium implants, increase wettability, and prevent bacterial adhesion [11].

**Figure 4:**
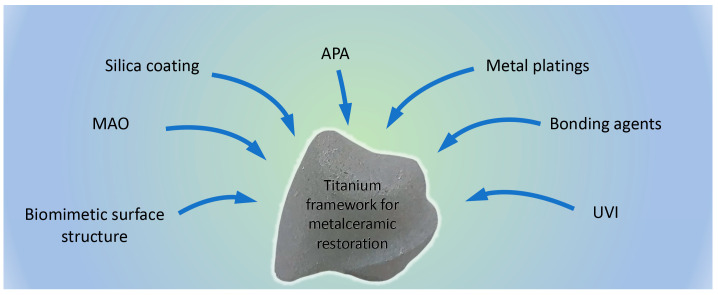
Treatment methods for improving bond strength between titanium frameworks and veneering ceramic material.

The control of thickness and structure of the surface oxide layer is challenging and crucial for the formation of the titanium–ceramic bond. Prior to ceramic veneering, different surface treatment methods may be applied, resulting in formation of either a controlled oxide layer with desired thickness and firmness, or an interlayer providing higher bond strength by adding specific elements on the interface between the alloy and the ceramic material. Metal–ceramic bond strength may be modified by different approaches, as shown in Figure 4 and Table 2.

Titanium–ceramic bond strength can be increased by several approaches:

– Mechanical surface treatment—sandblasting, airborne particle abrasion (APA)
– Physical surface treatment—laser treatment
– Chemical and electrochemical surface treatment—etching, anodization, microarc oxidation (MAO)
– Bonding agent application
– Biomimetic surface morphology creation
– Combined surface treatment

Titanium surface processing by sandblasting significantly improves the bond with the veneering ceramic material due to modifications of surface topography and creation of micromechanical retentions [47]. After sandblasting with Al_2_O_3_, SEM shows presence of aluminum particles on the titanium surface [31]. Attempts for removal of these particles by etching with combination of acids lead to a decrease in the metal–ceramic bond strength [48].

In their investigations Aslan et al. and Yuan et al. [49,50] studied the effect of coating agents on the metal–ceramic bond strength between milled titanium and porcelain. They found that coating with micro-arc oxidation (MAO) and hydroxyapatite significantly improved the bond compared to only airborne particle abrasion of titanium surfaces. Studies of Antanasova et al. showed that combination of surface treatment (APA) with Al_2_O_3_ and bonding agent application on both milled and additively manufactured specimens significantly increased the titanium–ceramic bond strength [51].

Studies of Vuorinen et al. revealed that plating silver on the titanium surface significantly improved the bond strength with the veneering ceramic material [52].

Hu et al. explored the effect of sandblasting pressure and the application of gold coating on titanium surfaces and reached the conclusion that both factors—increased pressure and coating—improved the bond between SLM titanium and ceramics [53]. In the same research they found that thermocycling did not affect titanium–ceramic bond strength.

Studies of Svanborg et al. showed that grinding prior to sandblasting might be excluded from the treatment protocol when a titanium framework was produced by additive manufacturing methods [54].

When tribochemical silica coating is applied to the titanium surface, silicon, which may be found within the titanium oxide layer, becomes involved in forming complex oxides. According to the studies of Fukuyama et al., these oxides improve the wettability of the metal surface, thus increasing both its compatibility with the bonding agent and titanium–ceramic bond strength [55].

Processing of the titanium surface with ultraviolet irradiation (UVI) increases the wettability and significantly improves the bond strength with porcelain without causing morphological changes on the treated areas [56].

Studies show that laser treatment with a ytterbium-doped fiber laser or an Nd:YAG laser may improve the bond strength between milled titanium and ceramics. However, sandblasting achieves higher values of adhesion [62].

Yang et al. demonstrated in their study that a biomimetic titanium surface with a strictly chosen design increases remarkably wettability and bond strength between ceramics and the titanium framework produced by selective laser melting [57].

On the other hand, Curtis et al. found that application of the coating agent provided by the producer of the ceramic system did not affect bond strength between milled titanium and ceramics [58].

Porcelain firing may also affect the titanium ceramic bond—the use of argon protective atmosphere during sintering results in a stronger bond compared to sintering in vacuum [59].

Airborne particle abrasion (APA, sandblasting) and application of bonding agents, which limit the formation of excessively thick and porous surface oxide layer, appear to be the most applied in everyday laboratory workflow. Although some of the other described methods provide similar or even better results, especially compared to bonding agent application, these two types of surface treatment are among the most popular ones probably because of their ease of application—they are cost-effective, do not require specific equipment and the working protocol for the specific bonding agent is given by the producers of the low fusing ceramics [63].

Studies showing that thermal and mechanical cycling may negatively affect the titanium ceramic bond, point to a matter of concern [60,61].

Although in-vitro investigations prove that titanium–ceramic bond strength meets the requirements of 25 MPa according to ISO 9693:2019, evidence of clinical performance is scarce. The study of Hey et al. investigated the survival rate of 45 titanium–ceramic crowns with CAD/CAM milled framework for a 6-year period of function [43]. It was found that neither loss of retention nor secondary caries lesion had occurred, indicating good marginal and internal fit of the restorations. The effect on the periodontal tissues was described as minimal, as no periodontal complications were present. However, 10 cohesive and 2 adhesive fractures were found during the studied period, resulting in 27% damaged crowns. Ceramic fractures are the major points of concern regarding titanium–ceramic fixed prosthetic restorations because they compromise esthetics and disturb function by loss of occlusal contacts and contacts to adjacent teeth.

## 5. Internal Fit, Marginal Fit

Internal and marginal fit of the final fixed prosthetic restorations are other important factors contributing to the longevity of dental treatment and to the occurrence of possible complications. Discrepancies in the marginal adaptation to the retainers may cause acute and chronic inflammation of the gingival tissues. Thick crown edges may serve as plaque-retentive spots. They may cause development of caries lesions on the abutments, destruction of hard dental tissues, tooth loss, and eventually treatment failure. Gurel et al. explored the marginal and internal fit of titanium and cobalt–chromium frameworks made by traditional casting and CAD/CAM milling [64]. They came to the conclusion that both types of tested materials and methods of production provide comparable and clinically acceptable fit of the dental restorations. Other studies show similar results, proving the possibility of wider use of titanium for fixed dental prostheses [65,66,67].

When comparing the fit of titanium crowns produced by the two innovative approaches—milling and selective laser melting, it was found that the additive technique provides a slightly better internal fit, though both are within the clinically accepted range [68,69].

Porcelain firing cycles may affect marginal and internal fit of titanium crowns by increasing the discrepancies between the restoration and the abutment [70].

## 6. Color and Esthetics

Methods for improving esthetics and masking the grayish color of titanium abutments and implant-supported frameworks include titanium anodization, providing enough thickness of the ceramic material, and choosing appropriate opacity of the cement [71,72]. According to the studies of Elter et al., airborne particle abrasion may increase the grayish color of the titanium surface, thus influencing the final esthetic result [73]. However, only the study of Korkmaz was available in the PubMed database regarding esthetic outcomes of titanium–ceramic crowns for the last 10 years. In this research three different techniques were used for production of the titanium frameworks of the specimens—milling of titanium grade 2, casting of titanium grade 1, and 3D printing (laser sintering) of Ti64 grade 5. Spectrophotometric color measurements were performed before opaque application, after opaque application, and after porcelain plus glaze application. Results showed that production method of the titanium framework may influence the final color and esthetic outcome, and it was concluded that casting and milling production methods provided better optical results than laser sintering [30].

## 7. Conclusions

Mechanical properties and biocompatibility of titanium and its alloys described in the last few decades allow them to replace successfully nickel– and cobalt–chromium base dental alloys. Although titanium is considered highly biocompatible, evidence reporting adverse effects can be found. This fact points out the necessity of future studies on the biological effects after long-term clinical exposure.

Titanium–ceramic prosthetic restorations appear to possess better biological response, similar fit, and comparable metal–ceramic bond strength compared to conventional metal–ceramics. However, there is still no uniform protocol for laboratory surface treatment of titanium.

Three-dimensional printing of titanium frameworks allows the creation of specific biomimetic surface morphology, thus increasing the wettability and metal–ceramic bond strength. The obstacles in the application of titanium in prosthodontics due to the specific requirements during casting have been overcome by the methods of subtractive and additive manufacturing. Novel CAD/CAM technologies have enabled the production of titanium frameworks with promising characteristics, which may be implemented widely in contemporary prosthetic treatment.

However, most of the presented studies are conducted in laboratory conditions with strictly defined experimental parameters. Further investigations are needed to assess the longevity, esthetics, and clinical behavior of titanium–ceramic fixed prosthetic restorations.

## Figures and Tables

**Table 1:** Characteristics of the most used titanium alloys in the dental field.

Type of Alloy	Microstructure	Advantages	Disadvantages	Indications of Use	Possible Production Techniques
Cp Ti grade 1	α	High biocompatibility, excellent resistance to corrosion, ductile	Lowest strength	Dental implants, implant abutments, prosthetic restoration frameworks	Casting, milling, 3D printing
Cp Ti grade 2	α	Good biocompatibility, good corrosion resistance,	Moderate strength	Dental implants, implant abutments, prosthetic restoration frameworks	Casting, milling, 3D printing
Cp Ti grade 3	α	Higher strength than grade 2, high biocompatibility	Lower ductility than grade 2	Dental implants, implant abutments, prosthetic restoration frameworks	Milling, casting, 3D printing
Cp Ti grade 4	α	Good strength, high biocompatibility	Lower ductility than grade 3	Dental implants, implant abutments, prosthetic restoration frameworks	Milling, 3D printing, casting
Ti6Al4V (grade 5)	α + β	High strength, good fatigue resistance	Presence of potentially toxic components (Al, V)	Dental implants, prosthetic frameworks	3D printing, milling, casting
Ti6Al4V ELI (grade 23)	α + β	High strength, good fatigue resistance	Presence of potentially toxic components (Al, V)	Dental implants, implant abutments, prosthetic frameworks	3D printing, casting, milling
Ti6Al7Nb	α + β	High strength, high biocompatibility	Presence of potentially toxic element (Al)	Dental implants, implant abutments, prosthetic frameworks	3D printing
Ti5Al2,5Fe (grade 9)	α + β	Good corrosion resistance, excellent biocompatibility, higher strength than cp Ti	Presence of potentially toxic element (Al)	Dental implants, implant abutments, prosthetic frameworks	3D printing, milling
Ti13Nb13Zr	β	Low elastic modulus, excellent biocompatibility	Complex processing	Dental implants	3D printing
Ti15Mo	β	High mechanical properties, good biocompatibility	Low wear resistance, surface treatment needed	Dental implants	3D printing

**Table 2:** Factors affecting titanium–ceramic bond strength.

	Authors	Type of Study	Method of Treatment/Specific Conditions	Type of Metal Alloy	Production Technique	Testing Method	Results
1	Zhao et al., 2016 [44].	In vitro	Applying bonding agent B_2_O_3_– La_2_O_3_-SrO–Na_2_O–Al_2_O_3_	Ti6Al4V	Selective laser melting (SLM)	Three point bending test	Increased metal–ceramic bond strength
2	Yang et al., 2016 [45].	In vitro	Bonding agent	Cp Ti grade 4	milling	Crack initiation stresses	Increased metal–ceramic bond strength
3	Zhang and Zhang 2015 [46].	In vitro	TiO(2)-SiO(2)-SnOx nano-coatings	Cp Ti	milling	Three-point flexure bond test	Increased metal–ceramic bond strength
4	Moldi et al., 2015 [47].	In vitro	Sandblasting with 250 μm Al_2_O_3_ particles	Cp Ti grade 2	casting	Fracture load by universal testing machine	Increased metal–ceramic bond strength
5	Papia et al., 2018 [31].	In vitro	Passivation/no passivation	CpTi grade 1 CpTi grade 2 Ti6Al4V	Casting Milling Electron beam melting (EBM)	Shear bond strength test using universal testing machine	Time of passivation does not affect bond strength Milled Ti showed lowest bond strength
6	Parchańska-Kowalik, Wołowiec-Korecka, and Klimek 2018 [48].	In vitro	Removal of Al_2_O_3_ particles after sandblasting by etching	Cp Ti grade 1	Casting	Shear bond strength	Decreased bond strength
7	Aslan, Ural, and Arici 2016 [49].	In vitro	Microarc oxidation (MAO) with hydroxyapatite (HA)	Titan grade 5	Milling	Bond strength by universal testing machine	Increased bond strength after coating with MAO and HA
8	Yuan et al., 2018 [50].	In vitro	MAO	Cp Ti grade 4	Milling	Three point bending test	Increased bond strength compared to only sandblasted titanium surface
9	Antanasova et al., 2020 [51].	In vitro	Airborne particle abrasion (APA) and bonding agent	Ti6Al4V Ti6Al4V	Milling SLM	Three point bending test	Increased bond strength No difference between milled and slm-produced specimens
10	Vuorinen et al., 2024 [52].	In vitro	Silver plating	Cp Ti grade 1	Milling	Three point bending test	Increased bond strength
11	Hu, Ren, and Luo 2023 [53].	In vitro	Sandblasting and gold plating	CpTi	SLM	Three point bending test	Increased bond strength
12	Svanborg et al., 2024 [54].	In vitro	Grinding and APA	TiAl6V4 ELI (Ti grade 23) Cp Ti grade 4	Additive manufacturing Milling	Shear bond strength test by universal testing machine	Grinding does not affect bond strength
13	Fukuyama, Hamano, and Ino 2016 [55].	In vitro	Silica-coating (silica containing alumina powder, Rocatec Plus, 3MESPE) and bonding agent	Cp Ti	Milling	Shear bond strength test by universal testing machine	Improved bond strength
14	Kumasaka et al., 2018 [56].	In vitro	Ultraviolet irradiation	Cp Ti gr 2	Milling	Tensile bond test	Improved bond strength
15	Yang, Liang, and Liu 2025 [57].	In vitro	Biomimetic surface design	Ti6Al4V (TC4)	Additive manufacturing	Bending tests in a universal testing machine	Surface structure design may affect titanium–ceramic bond strength
16	Curtis et al., 2015 [58].	In vitro	Vita Titankeramik’s bonding agent in powder, paste, and spray	Cp Ti grade 2	Milling	Three point bending test	Not affecting titanium–ceramic bond strength
17	Wang et al., 2020 [59].	In vitro	Porcelain firing in vacuum, argon or helium	Cp Ti grade 2	Milling	Three point bending test	Porcelain firing in argon atmosphere significantly improves bond strength
18	Antanasova et al., 2018 [60].	In vitro	Thermomechanical cycling	Cp Ti Ti6Al4V Ti6Al4V	Casting Milling SLM	Shear bond strength	Decreases bond strength
19	Yong and Bo 2016 [61].	In vitro	Thermomechanical cycling	Cp Ti Ti2448	Milling	Crack initiation test was performed using a universal testing machine	Decreases bond strength

## Abbreviations

APA	airborne particle abrasion
Cp Ti	commercially pure titanium
CAD/CAM	computer-aided design/computer-aided manufacturing
SLM	selective laser melting
SLS	selective laser sintering
EBM	electron beam melting
UVI	ultraviolet irradiation
MAO	micro-arc oxidation
HA	hydroxyapatite
Nd:YAG	neodymium-doped yttrium aluminum garnet
